# Effect of photon-induced photoacoustic streaming and shock-wave enhanced emission photoacoustic streaming technique on the removal of the smear layer after root canal preparation in curved root canals

**DOI:** 10.1016/j.jds.2022.06.019

**Published:** 2022-07-08

**Authors:** Jiaru Tong, Liu Liu, Jing Du, Yuan Gao, Dongzhe Song, Dingming Huang

**Affiliations:** a State Key Laboratory of Oral Diseases & National Clinical Research Center for Oral Diseases, West China Hospital of Stomatology, Sichuan University, Chengdu, China; b Department of Conservative Dentistry and Endodontics, West China Hospital of Stomatology, Sichuan University, Chengdu, China

**Keywords:** Root canal preparation, Smear layer, Root canal irrigation, Ultrasonic activation, Sonic activation, Laser-activated irrigation

## Abstract

**Background/purpose:**

The efficiency of root canal irrigation has an important impact on the prognosis of root canal treatment. Photon-induced photoacoustic streaming (PIPS) and shock wave enhanced emission photoacoustic streaming (SWEEPS) are the special modality of Er: YAG laser, whether can they improve the efficiency of root canal irrigation remains to be studied.

**Materials and methods:**

Fifty human teeth with curved root canals were collected and stored in the thymol solution until used in the study. After traditional endodontic cavities preparation, root canals were prepared to size #35 with a 0.04 taper. The final irrigating techniques were as follows: (I) manual dynamic activation (MDA), (II) ultrasonically activated irrigation (UAI), (III) sonically activated irrigation (SAI), (IV) PIPS, and (V) SWEEPS. Fifty teeth were randomly divided into five groups mentioned above. After root canal preparation, the roots were cleaved longitudinally. The dentine surfaces were photographed from the coronal, middle, and apical third of the root by scanning electron microscopy operated at a low vacuum. Two examiners separately graded each image according to the remained smear layer situations.

**Results:**

PIPS and SWEEPS groups showed fewer smear layers remaining than the others in the middle and the apical third (*P* *<* 0.05) of the root canal. In contrast, in the coronal third, five groups showed no significant difference (*P* > 0.05).

**Conclusion:**

PIPS and SWEEPS showed superior smear layer clearing efficiency than traditional irrigating techniques in curved root canals.

## Introduction

Bacterial and other microbial infections in the root canal system are the main pathogenic factors of dental pulp and periapical diseases. Root canal treatment (RCT) is the most effective and commonly used method for treating these diseases.^1^ The principle of RCT is to remove infectious substances in the root canal system and seal the root canal tightly to avoid re-infection of microorganisms, therefore curing the disease. The main steps of RCT include the opening of the pulp chamber, root canal preparation and root canal filling. Root canal preparation consists of root canal shaping and cleaning. Curvature in root canals will introduce complexity for instrumentation, and there is an increased risk of root canal surface areas remaining unprepared and uncleaned if the canal is curved.^2^ Therefore, it has been an agreement that the curvation of the root canals increases the difficulty of RCT.

The smear layer is an amorphous structure produced and adheres tenaciously to the root canal walls during root canal shaping.^3^ The smear layer could cover the surface of the dentine tubules, blocking the diffusion of irrigants, intracanal medication, and root canal sealers.^4^ As a result, the smear layer would affect the removal of infectious substances during root canal preparation, reducing the tightness between root canal filling materials and root canal wall, which may eventually affect the prognosis of RCT, or even lead to the treatment failure.^5^^,^^6^

A critical purpose of root canal cleaning is to remove the smear layer from the root canal system. The commonly used cleaning irrigants include sodium hypochlorite (NaClO), ethylenediaminetetraacetic acid (EDTA), chlorhexidine (CHX) and physiological saline. Studies suggested that alternation between EDTA and NaClO with appropriate cleaning techniques could remove the smear layer effectively.^7^^,^^8^

Laser is a technique introduced in dentistry in recent years due to its safety and effectiveness. At present, some kinds of lasers have been used in clinics, such as: Er: YAG laser, Nd: YAG laser, Er, Cr: YSGG laser, diode laser. Amongst them, Er: YAG laser is one of the most commonly used lasers in the treatment of dental pulp and periapical diseases, which has been used in the minimal removal of the dental pulp,^9^ root canal cleaning, and disinfection of bone cavity in endodontic microsurgery.^6^ Studies found that when used in root canal cleaning, Er: YAG laser can increase cleaning efficiency.^10^

Photon-induced photoacoustic streaming (PIPS) is a particular mode of Er: YAG. PIPS has a shorter pulse width than regular Er: YAG, which means PIPS can produce higher peak power under the same energy. It uses a 2940 nm Er: YAG laser with a stripped and conical fibre tip to pulse extremely low energy levels and transfers energy into the irrigant with only a minor increase in tooth temperature.^11^ It generates peak power spikes and intensive shock waves in water with minimal thermal effect, using low energy levels (10 or 20 mJ) and short pulse length (50 μsec).^12^ Some studies have examined this irrigating method's smear layer removal ability and have obtained different conclusions.^13^, ^14^, ^15^

Recently, a novel technique named shock-wave enhanced emission photoacoustic streaming (SWEEPS) has been developed and applied in clinics. SWEEPS replaces one short laser pulse (50 μsec) of PIPS with two ultrashort laser micro pulses (25 μsec), and there is an optimal delay time (generally 300–600 μsec) between them.^5^ The first micropulse generates the main bubble, and the second micropulse (occurring before the spontaneous collapse of the main bubble) increases the pressure to accelerate the collapse of the main bubble. The amplification of pressure waves by SWEEPS was greater than the standard PIPS irrigation procedure.^12^ The effectiveness of SWEEPS in removing the smear layer needs to be further studied.

This study aimed to assess laser-activated irrigation methods' smear layer clearing efficacy and compare them with traditional irrigating techniques in curved root canals. We hoped to provide suggestions for root canal irrigating treatment planning in clinics.

## Materials and methods

### Sample selection

Extracted human third molars were stored in distilled water with 1% thymol and then radiographed using a digital system (Sopro Acteon, Mérignac, France) in both mesiodistal and buccolingual directions. Third molars with at least one independent root canal were included. Those teeth presenting caries, resorptions, fractures, or history of teeth treatment were excluded. The root canal curvature was evaluated according to Schneider's evaluation method. Finally, 50 teeth with a slightly crooked root canal (20°< curvature < 40°) were included in this study. After cleaning with ultrasonic scalers and rinsing with distilled water, the teeth were stored in 0.05% thymol to prevent bacterial growth.

### Root canal preparation

Traditional access cavity preparation was used. The canal orifices were localised by scouting with a size 10 K-file (Dentsply Sirona, Ballaigues, Switzerland). Working length (WL) was determined as patency length minus 1 mm. For root canal preparation, a glide path was prepared with hand K-files size 10, .02 taper to size 15, .02 taper (Dentsply Sirona). The apical region of each root was covered externally with light-cured flowable resin (3M, St. Paul, MND, USA) to create a closed root canal system to obtain a vapor lock effect. Root canals were instrumented with rotary nickel-titanium instruments (S3 system, Sani, Shanghai, China) according to the manufacturer's recommendations to size 35, 0.04 taper. When changing files, the canals were irrigated with 1 ml 1% NaClO (Longly Biotechnology, Wuhan, China).

### Final irrigation

The final irrigation solutions were 1% NaClO, saline and 17% EDTA. The teeth were divided into five groups according to different cleaning techniques: (I) manual dynamic activation (MDA), (II) ultrasonically activated irrigation (UAI), (III) sonically activated irrigation (SAI), (IV) PIPS, (V) SWEEPS. Fifty teeth with curved root canals were randomly and evenly allocated to the above mentioned five groups. The protocol for final irrigation was established as follows.

1. 1% NaClO (5 mL, 1 min), activation for 30 s, resting phase 30 s, activation for 30 s.
2. Saline (5 mL), activation for 1 min.
3. EDTA (5 mL, 1 min), activation for 30 s, resting phase 30 s, activation for 30 s.
4. Saline (5 mL), activation for 1 min.

The operation steps of each cleaning technique were as follows:

(I) MDA: The 31-G side-cut open-ended needle (NaviTip, Ultradent, South Jordan, UT, USA) was used. The tip was inserted to 1 mm short of the WL and delivered with 2–3 mm amplitude back-and-forth movements. Irrigation was finished with manual up- and down-movement of the needle inside the root canal.
(II) UAI: a size 15, 0.02 taper ultrasonic tip (Irrisafe, Satelec Acteon, Mérignac, France) was inserted in the root canal within 2 mm of the WL. Irrigants were delivered into the access cavity with a 27-G needle during activation. The ultra-sound device was set at power 5 (Suprasson P5, Satelec Acteon).
(III) SAI: a sonic endo irrigation tip (Polyamide tip, VDW, Munich, Germany) was inserted in the root canal within 2 mm of the WL and activated with a frequency of 6000 Hz and an amplitude of 160 mm using an air scaler (KaVo Sonic-flex; KaVo, Biberach, Germany). Irrigants were delivered into the access cavity with a 27-G needle during activation.
(IV) PIPS: According to the manufacturer's recommendations, a 2940-nm wavelength Er: YAG laser equipped with a handpiece H14 was used (LightWalker AT, Fotona, Ljubljana, Slovenia). The laser parameters were 0.3 W, 15 Hz, 20 mJ, SSP mode, air/water turned off. The pulse length was 50 μm. A 9-mm-long and conical 600-μm fibre tip (PIPS 600/9, Fotona) was placed into the pulp chamber, kept stationary and did the activation.
(V) SWEEPS: A special auto SWEEPS Er: YAG laser modality and a special fibre tip (SWEEPS 600, Fotona) were used at a wavelength of 2940 nm Er: YAG laser (Fotona). The laser parameters were 0.3 W, 15 Hz, 20 mJ, SWEEPS mode, air/water turned off. The SWEEPS tip was placed into the pulp chamber and did the activation.

The root canals were dried using a suction cannula and paper points. All the procedures were performed by one operator.

### Scanning electron microscope (SEM) measurements and evaluations

After the above procedures, the teeth were separated into two parts along the long axis, followed by ethanol gradient elution, and sputter-coated with gold/palladium. The samples were examined with SEM (Oxford Instruments, London, UK) in the high-vacuum mode (10–20 kV, 5.0 mm working distance). One operator who did not know the experimental group randomly selected an area to take photos respectively from the coronal, middle and apical thirds of the root canal of each specimen at the magnification of 3000. Images were recorded digitally in lossless TIFF format.

The images were analysed blindly by two previously calibrated examiners. Intra- and inter-examiner reliability was verified (kappa test). Cleanliness was quantified using a 4-point scoring system adapted from Gambarini and Laszkiewicz and Kato et al.^16^^,^^17^ Score 1: opened dentinal tubules without debris; Score 2: opened dentinal tubules with debris covering less than 50% of the area; Score 3: opened dentinal tubules with debris covering more than 50% of the area; and Score 4: dentinal tubules covered by debris in 100% of the examined area (Fig. 2).

**Figure 1:**
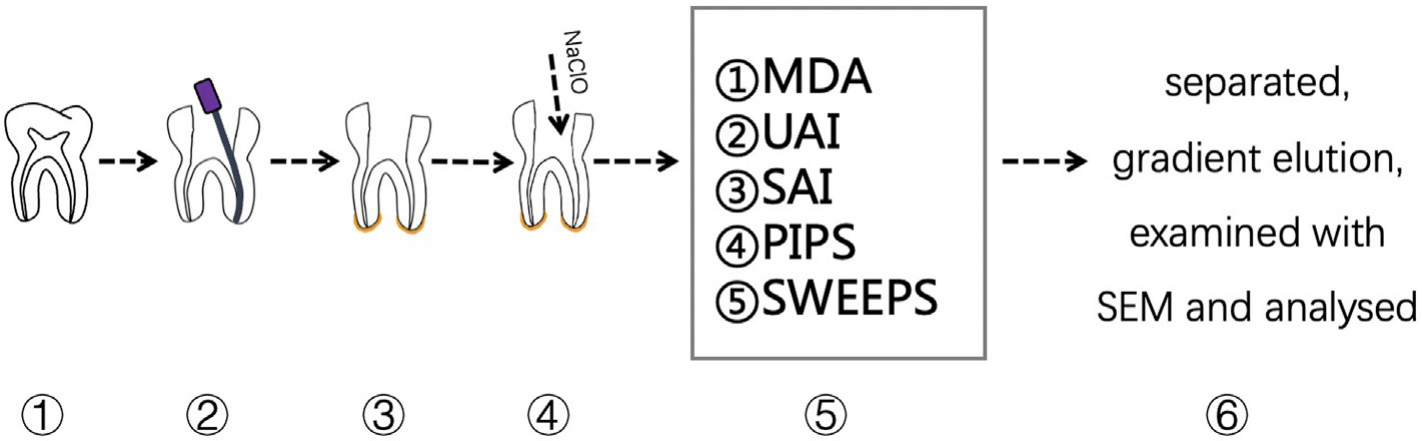
① open the pulp chamber; ② localize the canal orifices and measure the work length; ③ cover the apical region and mark the root canal chosen to do the irrigation; ④ root canal preparation; ⑤ root canal irrigation; ⑥ separate the teeth into two parts, ethanol gradient elution, and sputter-coated with gold/palladium, examine with SEM and analyse the images.

**Figure 2:**
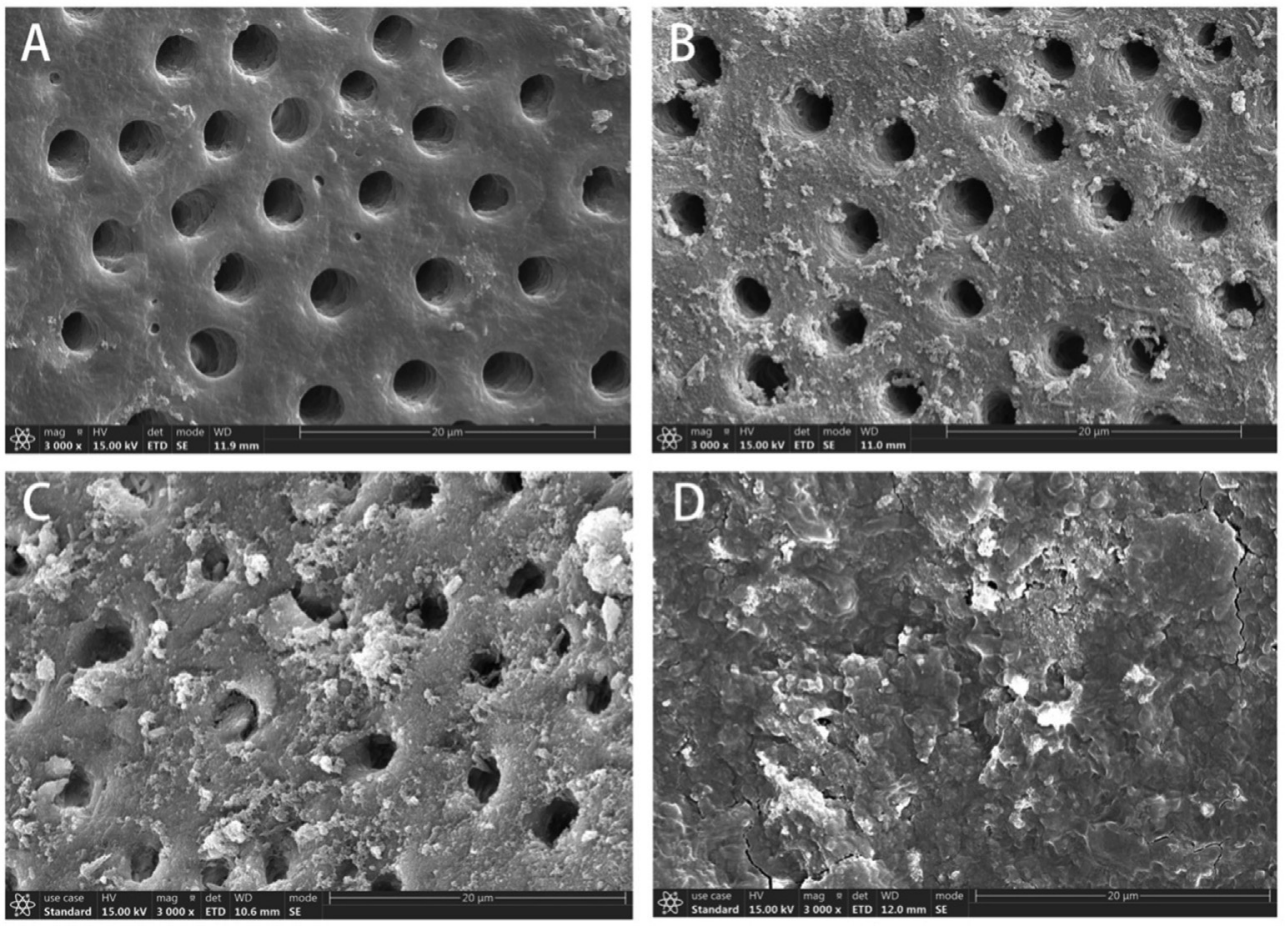
A (Score 1):opened dentinal tubules without debris; B (Score 2):opened dentinal tubules with debris covering less than 50% of the area; C (Score 3):opened dentinal tubules with debris covering more than 50% of the area; D (Score 4):dentinal tubules covered by debris in 100% of the examined area.

### Statistical analysis

The smear scores of each group were presented as mean rank scores and median scores in parentheses. The Shapiro-Wilk test was used to analyse the normality assumption of the data. Because the data were abnormally distributed (*P* < 0.05), the Kruskal-Wallis test (non-parametric test) was used to compare the differences among the groups. A post hoc comparison was performed using the Dunnett-T test when the Kruskal-Wallis test indicated a statistically significant difference. Statistical analysis was performed using the SPSS 26.0 software (IBM SPSS Inc., Armonk, NY, USA). The testing was performed at the 95% level of confidence (*P* < 0.05).

The workflow of the study is summarized in Fig. 1.

## Results

**Figure 3:**
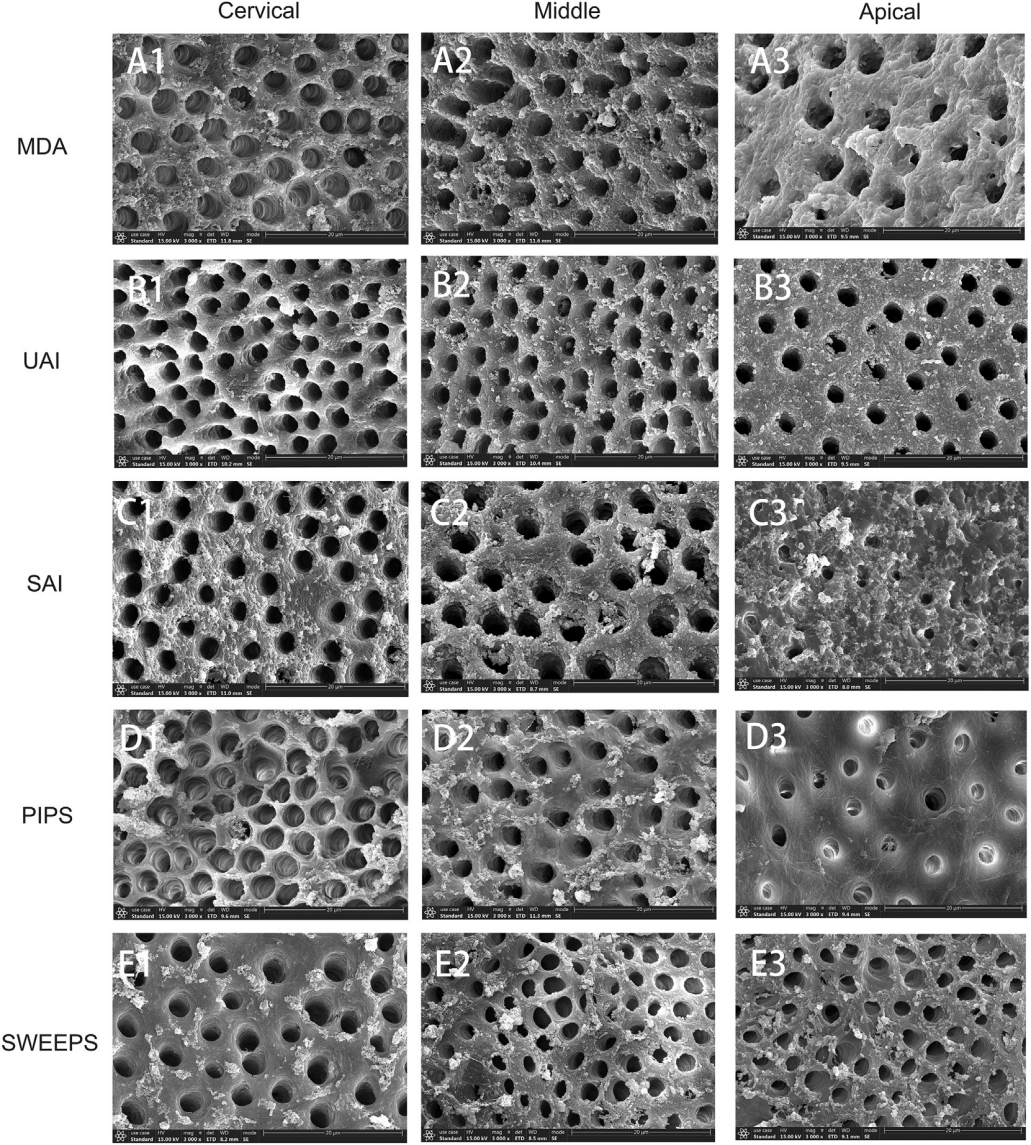
Coronal (1), middle (2) and apical (3) third of the five curved root canal groups (A–E). All images were recorded at 3000 times. Scale bars indicate 20 μm. MDA, manual dynamic activation. UAI, ultrasonic activated irrigation. SAI, sonically activated irrigation. PIPS, photon-induced photoacoustic streaming. SWEEPS, shock-wave enhanced emission photoacoustic streaming.

The SEM results of the smear layer remained on the root canal wall were shown in Fig. 3.

**Figure 4:**
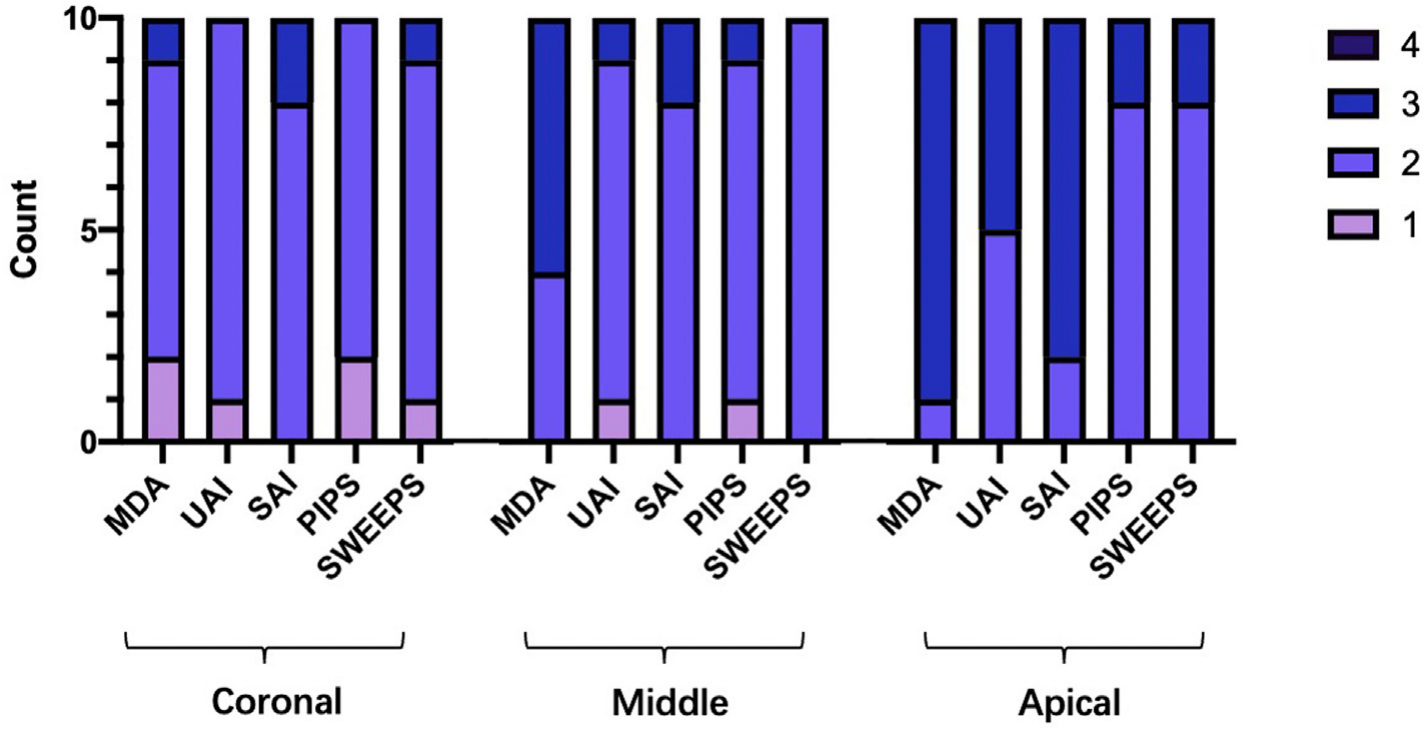
The distribution of scores for the smear layer according to the experimental groups. MDA, manual dynamic activation. UAI, ultrasonically activated irrigation. SAI, sonically activated irrigation. PIPS, photon-induced photoacoustic streaming. SWEEPS, shock-wave enhanced emission photoacoustic streaming.

The distribution of the scores of smear layer of the five groups were shown in Fig. 4. In the coronal and middle third of the root canal, the scores of smear layer in the five groups were most at score 2. In the apical third of the root canal, the scores of smear layer were mostly score 3 in the MDA, UAI and SAI groups, while in PIPS and SWEEPS groups were mostly score 2.

For the coronal third of the root canal, the smear layer scores of the five groups showed no significant difference between each other (*P* > 0.05), which indicated that the clearing efficiency of the five cleaning techniques was similar in this part.

For the middle third of the root canal, the MDA group's smear layer score was higher than the rest of the groups. However, significant difference only existed between SWEEPS and MDA (*P <* 0.05).

For the apical third of the root canal, scores of SWEEPS and PIPS were lower than SAI and MDA, with significant differences (*P <* 0.05). The scores meant that SWEEPS and PIPS had shown higher clearing efficiency of smear layer in the apical third of the root canal than MDA and SAI.

In all parts of the root canals, there was no significant difference between SWEEPS and PIPS (*P* > 0.05). It also showed that none of the root canals was completely free of smear layer, whatever the method of irrigant activation (Table 1).

**Table 1:** Mean rank scores, median of scores (in parentheses), and the results of the same root-thirds between the 5 groups.

	Coronal	Middle	Apical
MDA	24.1 (2.0)	35.9 (3.0)^a^	35.0 (3.0)^ab^
UAI	24.2 (2.0)	21.9 (2.0)	25.0 (2.5)
SAI	30.9 (2.0)	26.3 (2.0)	32.5 (3.0)^cd^
PIPS	21.9 (2.0)	21.9 (2.0)	17.5 (2.0)^bd^
SWEEPS	26.4 (2.0)	21.5 (2.0)^a^	17.5 (2.0)^ac^

The same lower case letter superscripts indicate significant difference (Dunnet-T test, P < 0.05) among groups within a root-third. MDA, manual dynamic activation. UAI, ultrasonically activated irrigation. SAI, sonically activated irrigation. PIPS, photon-induced photoacoustic streaming. SWEEPS, shock-wave enhanced emission photoacoustic streaming.

For the comparison amongst each root canal, in the PIPS and SWEEPS groups, there were no significant differences between the coronal, middle and apical third of the root canal (*P* > 0.05). In the other three groups, scores in the coronal part were lower than in the apical part, with significant differences (*P* < 0.05) (Table 2).

**Table 2:** Mean rank scores, median of scores (in parentheses), and the results after the statistical analysis using Kruskal-Wallis test and Dunnet-T Test by intra group comparison in the 5 groups.

	Coronal	Middle	Apical
MDA	8.5 (2.0)^ac^	16.9 (3.0)^c^	21.1 (3.0)^a^
UAI	12.3 (2.0)^a^	13.7 (2.0)	20.5 (2.5)^a^
SAI	12.5 (2.0)^a^	12.5 (2.0)^b^	21.5 (3.0)^ab^
PIPS	12.8 (2.0)	15.5 (2.0)	18.2 (2.0)
SWEEPS	14.6 (2.0)	14.5 (2.0)	17.4 (2.0)

The same lower case letter superscripts indicate significant difference (Dunnet-T test, P < 0.05) within a group among root-thirds. MDA, manual dynamic activation. UAI, ultrasonically activated irrigation. SAI, sonically activated irrigation. PIPS, photon-induced photoacoustic streaming. SWEEPS, shock-wave enhanced emission photoacoustic streaming.

## Discussion

Eick et al. first reported the smear layer in 1970, using the electron microprobe with SEM.^18^ It contained bacteria, bacteria by-products and necrotic tissue.^19^ Also, the smear layer might act as a substrate for bacteria, allowing more profound penetration into the dentinal tubules.^20^ So, it is crucial to remove the smear layer during root canal cleaning. EDTA and NaClO could effectively remove the smear layer's inorganic and organic components.^8^^,^^21^ The combination of the two had a good effect on removing the smear layer.^22^, ^23^, ^24^ The efficiency of the irrigants was also closely related to the irrigating techniques and methods; therefore, this study aimed to compare the clearing efficiency of the smear layer of five irrigant activation techniques in root canal cleaning.

Canal curvature and apical diameter influence the efficacy of root canal cleaning. Irrigation is significantly less effective in curved canals with a small apical diameter than in those with a larger apical diameter.^25^ Elnaghy et al. investigated the effectiveness of different activation systems in curved root canals. They stated that SAI debris removal was significantly more effective in the coronal region than in the apical part.^26^ Rödig et al. found that in curved root canals, activation of NaClO and EDTA with UAI resulted in significantly more effective smear layer removal in the coronal third of the root canal compared with no agitation.^27^ The effectiveness of irrigating techniques in canals with moderate to severe curvatures needs further investigation.^28^ Moreover, the irrigating effectiveness of the newly introduced PIPS and SWEEPS in curved root canals was not known.

Commonly used irrigating activation techniques are manually double SidePort needles-and-syringes, ultrasonic activated technique, and sonic activated technique. When the irrigation needle is inserted into a closed-end canal, there will be a space below the solution during irrigating because of air entrapment. This phenomenon is called the vapour lock,^29^ which could reduce the smear layer clearing efficiency.^30^ Ultrasonically activated irrigation (UAI) and sonically activated irrigation (SAI) are used to release the vapour lock.^31^^,^^32^ Ultrasonic working tip generates micro acoustic flow through high-frequency vibration (>25 kHz). The micro acoustic flow could relieve the vapour lock, transport the irrigants to the complex areas of the root canal, increase shear stress on the dentin wall and produce a mechanical scouring effect on the root canal wall to remove the smear layer.^33^^,^^34^ Sonic devices work similarly but generally use flexible tips and operate at lower frequencies.^35^ Studies showed that none of these traditional methods could remove the smear layer thoroughly, so some new techniques have been developed and researched.

Er:YAG laser is a near-infrared laser with a wavelength of 2.94 μm, similar to the peak absorption value of water (2.95 μm).^36^ When putting a conical-ended laser fibre into the pulp cavity, the absorption of the laser energy would induce shock waves into the irrigant and increase the cleaning efficiency.^37^ PIPS, as a special mode of Er: YAG, generates peak power spikes and intensive shock waves in water with minimal thermal effect, using low energy levels (10 or 20 mJ) and short pulse length (50 μsec).^12^ Under 20 mJ/0.3w, PIPS could effectively remove the smear layer without damaging the dentin tissue.^13^ Gordon et al.^15^ have found that compared with manually needle-and-syringe irrigation, PIPS could more effectively clean the root canal wall and obtain more open dentin tubules. However, some studies found no differences between PIPS and manual techniques. Stamatina has examined the irrigation effect of PIPS and found no significant difference compared with manually needle-and-syringe irrigation.^14^

SWEEPS is a novel technique which places a laser fibre tip in the access cavity filled with irrigants and emits a pulsed laser light into the fluid. SWEEPS has been developed primarily to improve the cleaning and disinfecting efficacy of the PIPS procedure.^12^ The operation of SWEEPS is similar to PIPS, but the mode of action is different in that SWEEPS delivers pulse pairs into the liquid.^38^ The amplification of pressure waves using SWEEPS was more significant than the standard PIPS irrigation procedure, which emitted a single Er: YAG pulse.^12^ Galler et al. found that PIPS had the deepest penetration depth of the irrigants while SWEEPS did not show benefits compared even to MDA.^35^ So, the smear layer clearing efficiency of PIPS and SWEEPS needs to be studied.

In this study, there was no significant difference between the five methods in the coronal third of the root canal. In this area, the root canal was straight and open wide after shaping, so the irrigants could fully contact the root canal wall without affecting the vapour lock. Mayer et al.^39^ also found that only the coronal third of the root canal was cleaned when irrigated with ultrasonic, and no difference in other areas.

In the middle third of the root canal, SWEEPS showed the best clearing ability of smear layers, while the other four groups were similar to each other. Gordon et al.^15^ found that PIPS could more effectively clean the root canal walls and obtain more open dentine tubules in a straight root canal than manually needle-and-syringe irrigation. Wang et al.^40^ had the same conclusion. In this study, PIPS showed no significant differences with MDA, UAI and SAI groups. However, we found that SWEEPS enhanced the irrigating efficiency significantly. This suggests that SWEEPS might have a better clearing ability of smear layers than the other four cleaning techniques.

In the apical third of the root canal, PIPS and SWEEPS showed the best clearing efficiency of smear layers, and there was no significant difference between PIPS and SWEEPS. For MDA group, the curvation might affect the tip placement and prevent the complete contact between irrigants and the root canal wall. As for UAI and SAI groups, increased wall contact of the tip with the root canal walls could result in no cavitation effects and reduce the clearing efficiency of the smear layer.^28^^,^^41^ Tember et al.^21^ also found that ultrasonic tip placed within 1 mm of the apical foramen did not show higher efficacy in smear layer removal than the traditional irrigation. However, in PIPS and SWEEPS groups, the fibre tip was placed into the pulp cavity and stayed statically, the placement of the tip was not affected by the curvation of the root canal, so the laser pulse produced at the laser fibre tip would not be influnced. Based on the result, we could infer that the irrigants could be activated and act on the root canal in the apical part of the curved root canals. Harry et al.^29^ also concluded that compared with UAI, laser activation could remove the vapour lock more effectively from the apical third of the root canal. For these reasons, the smear layer clearing efficiency of PIPS and SWEEPS might be the greatest in the apical third of the root canal.

There was no significant difference between the three parts of the root canal in the PIPS and SWEEPS groups in each root canal. In contrast, the clearing efficiency was higher in the coronal part than in the apical part, with significant differences in MDA, UAI and SAI groups. In these groups, the curvation could block the tip placement; the activated instrument contacts the apical third of the root canal wall and cannot produce cavitation or acoustic microstreaming, thus reduced the clearing efficiency. However, the tip placement of the laser groups was not affected by the root canal curvation, so the power and intensity of the shock wave were not affected. So, except for the two laser-activated irrigation groups, the smear layer efficiency was better in the coronal third than in the apical third in the manual, UAI, and SAI groups. But whether it can produce bubble collapse of the same intensity at all positions of curved root canals remains to be further verified.

PIPS and SWEEPS showed superior smear layer removal in the apical third of the curve root canal compared to the other irrigation strategies. However, the smear layer still cannot be removed entirely. With the continuous popularisation of lasers, they might have a better application prospect in root canal cleaning because of their excellent ability of smear layer clearing and simple operation compared with the existing methods.

## Declaration of Competing Interest

The authors have no conflicts of interest relevant to this article.

## References

[1] Siqueira Junior J.F., Rocas I.D.N., Marceliano-Alves M.F., Perez A.R., Ricucci D. (2018). Unprepared root canal surface areas: causes, clinical implications, and therapeutic strategies. Braz Oral Res.

[2] Roane J.B., Sabala C.L., Duncanson M.G. (1985). The "balanced force" concept for instrumentation of curved canals. J Endod.

[3] Mader C.L., Baumgartner J.C., Peters D.D. (1984). Scanning electron microscopic investigation of the smeared layer on root canal walls. J Endod.

[4] Foster K.H., Kulild J.C., Weller R.N. (1993). Effect of smear layer removal on the diffusion of calcium hydroxide through radicular dentin. J Endod.

[5] Jezersek M., Jereb T., Lukac N., Tenyi A., Lukac M., Fidler A. (2019). Evaluation of apical extrusion during novel Er:YAG laser-activated irrigation modality. Photobiomodul Photomed Laser Surg.

[6] Kadic S., Baraba A., Miletic I. (2020). Influence of different laser-assisted retrograde cavity preparation techniques on bond strength of bioceramic-based material to root dentine. Laser Med Sci.

[7] Violich D.R., Chandler N.P. (2010). The smear layer in endodontics - a review. Int Endod J.

[8] Baumgartner J.C., Ibay A.C. (1987). The chemical reactions of irrigants used for root canal debridement. J Endod.

[9] Wang M., Ma L., Li Q., Yang W. (2020). Efficacy of Er:YAG laser-assisted direct pulp capping in permanent teeth with cariously exposed pulp: a pilot study. Aust Endod J.

[10] Macedo R.G., Wesselink P.R., Zaccheo F., Fanali D., Van Der Sluis L.W. (2010). Reaction rate of NaOCl in contact with bovine dentine: effect of activation, exposure time, concentration and pH. Int Endod J.

[11] Lloyd A., Uhles J.P., Clement D.J., Garcia-Godoy F. (2014). Elimination of intracanal tissue and debris through a novel laser-activated system assessed using high-resolution micro-computed tomography: a pilot study. J Endod.

[12] Yang Q., Liu M.W., Zhu L.X., Peng B. (2020). Micro-CT study on the removal of accumulated hard-tissue debris from the root canal system of mandibular molars when using a novel laser-activated irrigation approach. Int Endod J.

[13] DiVito E., Peters O.A., Olivi G. (2012). Effectiveness of the erbium:YAG laser and new design radial and stripped tips in removing the smear layer after root canal instrumentation. Laser Med Sci.

[14] Passalidou S., Calberson F., De Bruyne M., De Moor R., Meire M.A. (2018). Debris removal from the mesial root canal system of mandibular molars with laser-activated irrigation. J Endod.

[15] Gordon W., Atabakhsh V.A., Meza F. (2007). The antimicrobial efficacy of the erbium, chromium:yttrium-scandium-gallium-garnet laser with radial emitting tips on root canal dentin walls infected with Enterococcus faecalis. J Am Dent Assoc.

[16] Gambarini G., Laszkiewicz J. (2002). A scanning electron microscopic study of debris and smear layer remaining following use of GT rotary instruments. Int Endod J.

[17] Kato A.S., Cunha R.S., da Silveira Bueno C.E., Pelegrine R.A., Fontana C.E., de Martin A.S. (2016). Investigation of the efficacy of passive ultrasonic irrigation versus irrigation with reciprocating activation: an environmental scanning electron microscopic study. J Endod.

[18] Eick J.D., Wilko R.A., Anderson C.H., Sorensen S.E. (1970). Scanning electron microscopy of cut tooth surfaces and identification of debris by use of the electron microprobe. J Dent Res.

[19] Yamada R.S., Armas A., Goldman M., Lin P.S. (1983). A scanning electron microscopic comparison of a high volume final flush with several irrigating solutions: part 3. J Endod.

[20] George S., Kishen A., Song K.P. (2005). The role of environmental changes on monospecies biofilm formation on root canal wall by Enterococcus faecalis. J Endod.

[21] Schmidt T.F., Teixeira C.S., Felippe M.C., Felippe W.T., Pashley D.H., Bortoluzzi E.A. (2015). Effect of ultrasonic activation of irrigants on smear layer removal. J Endod.

[22] Teixeira C.S., Felippe M.C., Felippe W.T. (2005). The effect of application time of EDTA and NaOCl on intracanal smear layer removal: an SEM analysis. Int Endod J.

[23] Kuah H.G., Lui J.N., Tseng P.S., Chen N.N. (2009). The effect of EDTA with and without ultrasonics on removal of the smear layer. J Endod.

[24] Lui J.N., Kuah H.G., Chen N.N. (2007). Effect of EDTA with and without surfactants or ultrasonics on removal of smear layer. J Endod.

[25] Nguy D., Sedgley C. (2006). The influence of canal curvature on the mechanical efficacy of root canal irrigation in vitro using real-time imaging of bioluminescent bacteria. J Endod.

[26] Elnaghy A.M., Mandorah A., Elsaka S.E. (2017). Effectiveness of XP-endo Finisher, EndoActivator, and File agitation on debris and smear layer removal in curved root canals: a comparative study. Odontology.

[27] Rodig T., Dollmann S., Konietschke F., Drebenstedt S., Hulsmann M. (2010). Effectiveness of different irrigant agitation techniques on debris and smear layer removal in curved root canals: a scanning electron microscopy study. J Endod.

[28] Neelakantan P., Ounsi H.F., Devaraj S., Cheung G.S.P., Grandini S. (2019). Effectiveness of irrigation strategies on the removal of the smear layer from root canal dentin. Odontology.

[29] Peeters H.H., Gutknecht N. (2014). Efficacy of laser-driven irrigation versus ultrasonic in removing an airlock from the apical third of a narrow root canal. Aust Endod J.

[30] Leoni G.B., Versiani M.A., Silva-Sousa Y.T., Bruniera J.F., Pecora J.D., Sousa-Neto M.D. (2017). Ex vivo evaluation of four final irrigation protocols on the removal of hard-tissue debris from the mesial root canal system of mandibular first molars. Int Endod J.

[31] Macedo R.G., Verhaagen B., Fernandez R.D. (2014). Sonochemical and high-speed optical characterization of cavitation generated by an ultrasonically oscillating dental file in root canal models. Ultrason Sonochem.

[32] Huffaker S.K., Safavi K., Spangberg L.S., Kaufman B. (2010). Influence of a passive sonic irrigation system on the elimination of bacteria from root canal systems: a clinical study. J Endod.

[33] Jiang L.M., Verhaagen B., Versluis M., Langedijk J., Wesselink P., van der Sluis L.W. (2011). The influence of the ultrasonic intensity on the cleaning efficacy of passive ultrasonic irrigation. J Endod.

[34] Bryce G., MacBeth N., Gulabivala K., Ng Y.L. (2018). The efficacy of supplementary sonic irrigation using the EndoActivator system determined by removal of a collagen film from an ex vivo model. Int Endod J.

[35] Galler K.M., Grubmuller V., Schlichting R. (2019). Penetration depth of irrigants into root dentine after sonic, ultrasonic and photoacoustic activation. Int Endod J.

[36] Do Q.L., Gaudin A. (2020). The efficiency of the Er: YAG laser and photonInduced photoacoustic streaming (PIPS) as an activation method in endodontic irrigation: a literature Review. Laser Med Sci.

[37] George R., Meyers I.A., Walsh L.J. (2008). Laser activation of endodontic irrigants with improved conical laser fiber tips for removing smear layer in the apical third of the root canal. J Endod.

[38] Lukac N., Jezersek M. (2018). Amplification of pressure waves in laser-assisted endodontics with synchronized delivery of Er:YAG laser pulses. Laser Med Sci.

[39] Mayer B.E., Peters O.A., Barbakow F. (2002). Effects of rotary instruments and ultrasonic irrigation on debris and smear layer scores: a scanning electron microscopic study. Int Endod J.

[40] Wang X., Cheng X., Liu B., Liu X., Yu Q., He W. (2017). Effect of laser-activated irrigations on smear layer removal from the root canal wall. Photomed Laser Surg.

[41] Ahmad M., Pitt Ford T.R., Crum L.A., Walton A.J. (1988). Ultrasonic debridement of root canals: acoustic cavitation and its relevance. Int Endod J.

